# To: Clinical outcomes of intensive care unit-acquired weakness in critically ill COVID-19 patients. A prospective cohort study

**DOI:** 10.62675/2965-2774.20240218-en

**Published:** 2024-10-31

**Authors:** Rohan Magoon, Varun Suresh, Nitin Choudhary

**Affiliations:** 1 Atal Bihari Vajpayee Institute of Medical Sciences Department of Anaesthesia New Delhi India Department of Anaesthesia, Atal Bihari Vajpayee Institute of Medical Sciences - New Delhi, India.; 2 Sheikh Jaber Al-Ahmad Al-Sabah Hospital Department of Anesthesia and Intensive Care Kuwait Department of Anesthesia and Intensive Care, Sheikh Jaber Al-Ahmad Al-Sabah Hospital - Kuwait - Arabian Gulf.; 3 All India Institute of Medical Sciences Pain Medicine and Critical Care Department of Anaesthesiology New Delhi India Department of Anaesthesiology, Pain Medicine and Critical Care, All India Institute of Medical Sciences - New Delhi, India.

To the Editor

In a thought-provoking study, Werlang et al. prospectively evaluated the clinical outcomes of critically ill patients with coronavirus disease 2019 (COVID-19) complicated by intensive care unit-acquired weakness (ICUAW).^(1)^ Citing the risk factors for neurological complications, the authors suggest similarities with regard to the predisposition to ICUAW between COVID-19 and non-COVID-19 cohorts.^(1,2)^ In this context, we draw attention of the readership to relevant studies on the subject, highlighting a facet that was overlooked in the index study.

Wolfe et al. studied the impact of vasoactive medications on ICUAW in mechanically ventilated patients and obtained interesting results.^(2)^ The use of vasoactive drugs in their setting increased the propensity to develop ICUAW, with an odds ratio (OR) and 95% confidence interval (95%CI) of 3.2 and 1.29 - 7.95, respectively (p value of 0.01) obtained with logistic regression analysis, independent of other established risk factors for weakness in critically ill patients. Notably, for every additional day that an ICU patient received a vasoactive agent, the odds of progressing to ICUAW increased by 35% (OR: 1.35; 95%CI: 1.1 - 1.65, p = 0.004). Moreover, when the role of the cumulative vasopressor dose was assessed, every 1µg/kg/d of norepinephrine administered resulted in a 1% increased odds of developing ICUAW (OR 1.01; 95%CI 1.001 - 1.02, p = 0.04), indicating a dose-dependent response.^(2)^

Even limiting the analysis to the use of vasopressors in patients with COVID-19, observational clinical studies have revealed that 35 - 94% of critically ill COVID-19 patients required hemodynamic support with vasopressors, with a weighted average of 66%.^(3)^ Moreover, independent researchers such as Cavalleri et al., who have studied the 1-yr functional decline in COVID-19 and non-COVID-19 survivors, suggest that there is a prolonged duration of vasopressor support in the subset of critically ill patients with COVID-19 compared with the duration in the subset without COVID-19.^(4)^ In addition, the data on vasopressor support in a recent systematic review and meta-analysis by Mermiri et al. on the greater prognostic implications of the use of vasopressors in the context of COVID-19 could elucidate the findings of Werlang et al., who evaluated the prognosis of ICUAW, and highlights the importance of these observations to current research.^(1,5)^
