# Supplementary material for: To: Clinical outcomes of intensive care unit-acquired weakness in critically ill COVID-19 patients. A prospective cohort study
Source: Crit Care Sci. 2024 Oct 31;36:e20240218en. doi: 10.62675/2965-2774.20240218-en (PMC11634286; doi:10.62675/2965-2774.20240218-en)
Supplement: Translation

# Translation: Para: Desfechos clínicos da fraqueza adquirida na unidade de terapia intensiva em pacientes com COVID-19 em estado crítico. Estudo de coorte prospectivo

**DOI:** 10.62675/2965-2774.20240218-pt

**Authors:** Rohan Magoon, Varun Suresh, Nitin Choudhary

**Affiliations:** 1 New Delhi India Department of Anaesthesia, Atal Bihari Vajpayee Institute of Medical Sciences - New Delhi, India.; 2 Kuwait Department of Anesthesia and Intensive Care, Sheikh Jaber Al-Ahmad Al-Sabah Hospital - Kuwait - Arabian Gulf.; 3 New Delhi India Department of Anaesthesiology, Pain Medicine and Critical Care, All India Institute of Medical Sciences - New Delhi, India.

Ao Editor

Em um estudo instigante, Werlang et al. avaliaram prospectivamente os desfechos clínicos de pacientes críticos com a doença do coronavírus 2019 (COVID-19) agravada por fraqueza adquirida na unidade de terapia intensiva (FAUTI).^(1)^ Ao citar os fatores de risco de complicações neurológicas, os autores sugerem semelhanças quanto à predisposição à FAUTI entre as coortes com e sem COVID-19.^(1,2)^ Nesse contexto, chamamos a atenção dos leitores para estudos relevantes sobre o assunto, destacando uma faceta que foi negligenciada no estudo índice.

Wolfe et al. estudaram o impacto de medicamentos vasoativos na FAUTI em pacientes ventilados mecanicamente e obtiveram resultados interessantes.^(2)^ O uso de medicamentos vasoativos em seu ambiente aumentou a propensão de desenvolver FAUTI, com uma razão de chances (RC) e um intervalo de confiança de 95% (IC95%) de 3,2 e 1,29 - 7,95, respectivamente (valor de p de 0,01) obtidos com a análise de regressão logística, independentemente de outros fatores de risco estabelecidos para fraqueza em pacientes críticos. Notavelmente, para cada dia adicional em que um paciente da UTI recebeu um agente vasoativo, as chances de progredir para a FAUTI aumentaram 35% (RC de 1,35; IC95% 1,1 - 1,65; p = 0,004). Além disso, quando o papel da dose cumulativa de vasopressor foi avaliado, cada 1µg/kg/d de norepinefrina administrada resultou em aumento de 1% na chance de desenvolver FAUTI (RC 1,01; IC95% 1,001 - 1,02; p = 0,04), indicando uma resposta dependente da dose.^(2)^

Mesmo limitando a análise ao uso de vasopressores em pacientes com COVID-19, estudos clínicos observacionais revelaram que 35 a 94% dos pacientes críticos com COVID-19 precisaram de suporte hemodinâmico com vasopressores, com média ponderada de 66%.^(3)^ Além disso, pesquisadores independentes, como Cavalleri et al., que estudaram o declínio funcional em 1 ano em sobreviventes e não sobreviventes da COVID-19, sugerem que há uma duração prolongada de suporte vasopressor no subgrupo de pacientes graves com COVID-19 em comparação com a duração no subgrupo sem COVID-19.^(4)^ Além disso, os dados de suporte vasopressor, numa recente revisão sistemática e metanálise de Mermiri et al. sobre as maiores implicações prognósticas do uso de vasopressores no contexto da COVID-19, poderiam elucidar os achados de Werlang et al., que avaliaram o prognóstico da FAUTI e destacam a importância dessas observações em estudos atuais.^(1,5)^
